# Changes in diet through adolescence and early adulthood: longitudinal trajectories and association with key life transitions

**DOI:** 10.1186/s12966-018-0719-8

**Published:** 2018-09-10

**Authors:** Eleanor M. Winpenny, Esther M. F. van Sluijs, Martin White, Knut-Inge Klepp, Bente Wold, Nanna Lien

**Affiliations:** 1 Centre for Diet and Activity Research & MRC Epidemiology Unit, School of Clinical Medicine, University of Cambridge, Cambridge, UK; 2 Department of Nutrition, Faculty of Medicine, University of Oslo, Oslo, Norway; 3 Department of Health Promotion and Development, Faculty of Psychology, University of Bergen, Bergen, Norway

**Keywords:** Diet, Vegetable, Fruit, SSB, Confectionery, Adolescence, Longitudinal, Cohort, Transition

## Abstract

**Background:**

Early adulthood is a period associated with poor diet and rapid weight gain. This is also an age of transition, including environmental, social and lifestyle changes which may be associated with changes in diet. We assess longitudinal associations between four early adulthood life transitions (leaving home, leaving education, entering employment, and cohabitation) and changes in consumption of fruit, vegetables, confectionery and sugar-sweetened beverages (SSBs).

**Methods:**

Participants (*n* = 1100) from the Norwegian Longitudinal Health Behaviour Study, reported data on diet and life transitions on up to eight occasions from age 14 to age 30. Diet data were self-reported in response to questions on intake of fruit, vegetables, confectionery and sugar-sweetened beverages. Growth models were developed to describe changing intake of each of the four diet indicators with age. Fixed-effects regression models assessed associations between the four life transitions and within-individual changes in diet indicators, with adjustment for the remaining transitions and parenthood.

**Results:**

Diet indicators showed quadratic trajectories with age: fruit and vegetable intakes declined from age 14 to ages 23 and 21 respectively, before increasing to age 30. SSB and confectionery intakes increased to age 18, before subsequently decreasing. Leaving the parental home was associated with a decrease in fruit intake of − 0.54 times/week (95% confidence interval (95%CI): -0.87;-0.22) and vegetable intake of − 0.43 times/week (95%CI: -0.70;-0.15). Leaving education was associated with increases in confectionery (0.33 times/week (95%CI: 0.04;0.62)) and SSB intakes (0.49 times/week (95%CI: 0.10;0.87).

**Conclusions:**

Leaving home and leaving education are associated with negative changes in diet and may present opportunities for effective diet and obesity intervention. Further study of these transitions is needed to understand the mechanisms mediating associations between life transitions and changes in diet.

**Electronic supplementary material:**

The online version of this article (10.1186/s12966-018-0719-8) contains supplementary material, which is available to authorized users.

## Background

Poor diet during adolescence and early adulthood contributes towards the rapid increases in weight gain seen during this period [1, 2], as well as to increases in other cardiometabolic risk factors [3]. Furthermore, poor dietary behaviours developed in childhood may persist into adulthood [4], influencing risk of non-communicable disease in later life [5–7]. Better understanding of factors affecting changes in diet and the establishment of long-term dietary behaviours during adolescence and early adulthood are required to underpin the development and targeting of public health interventions.

Late adolescence to early adulthood is a period of life transition [8, 9] which may allow disruption of an individuals’ pre-existing habits and allow changes in diet and dietary behaviours [10, 11]. Transitions that frequently occur during this period include: leaving the parental home, leaving school to begin further education or paid employment, and formation of partner relationships, including marriage, leading to cohabitation.

Previous research showing associations of diet with the home environment [12–15], employment status [16] and relationship status [17, 18] have primarily been cross-sectional. For example, cross-sectional studies have demonstrated associations between adolescent diet quality and factors related to the family home environment, such as associations between availability of particular foods (e.g. fruit and vegetables and soft drinks) in the home and adolescent intake of those foods, associations between parental and adolescent diet, and association of family meals with higher adolescent diet quality [12–15, 19, 20]. Among university students, a study across four European countries found that those living in the parental home consumed more fruit and vegetables than students living elsewhere [21]. In the US, studies have demonstrated associations between workplace environments (availability of unhealthy foods and co-worker attitudes to a healthy diet) and diet in young adults [16], and workplace environments may differ considerably from previous school or university environments where there is strong evidence for associations between school environment and health behaviours [22–24].

Studies examining associations between relationship status and health behaviours have shown mixed results. A cross-sectional study from the US found no associations between relationship status and fruit and vegetable, sugar-sweetened beverages (SSB) or fast food intake frequency among young adults [17], while a longitudinal study of young Australians found no differences in change in diet quality between those who began partnership relationships and those who did not [25]. Meanwhile in a Swiss adult sample, associations were seen between cohabitation and higher vegetable intake particularly among males [18].

To understand the impact of early adulthood transitions on changes in diet over time, analysis of longitudinal datasets is needed. In this study we investigated associations between diet indicators (fruit, vegetables, SSBs and confectionery) and key life transitions: leaving home, leaving education, entering employment, and cohabitation. We aimed to answer the question: how are each of these life transitions independently associated with longitudinal changes in diet?

## Methods

### Study overview and data collection procedures

The Norwegian Longitudinal Health Behaviour Study (NLHBS) focuses on health behaviour, lifestyle, and self-reported health from adolescence into adulthood. The study was approved by the Norwegian Data Inspectorate and has been conducted in full accordance with ethical principles, including the provisions of the World Medical Association Declaration of Helsinki. Information was collected by self-completion questionnaire with core questions repeated at every survey wave.

In autumn 1990, all 13-year-olds (*n* = 1195) in 22 schools randomly selected from Hordaland County, Norway, were invited to participate in the study. Written consent from parents/guardians and participants was required prior to participation. Response rate at baseline was 77.6% (*n* = 924) and no differences were found between participants and non-participants at baseline [26]. New students enrolling in participating schools in the first 2 years of data collection were invited to join the study, increasing the total sample size to 1134 [27]. Data were collected from participants nine times, at ages 13, 14, 15, 16, 18, 19, 21, 23 and 30. Questionnaires were completed at school from ages 13 to 15, and participants received questionnaires by post thereafter. Participants who participated in the survey at least once from age 14 to age 30 were included in the descriptive element of this analysis. Participants included in the transition analyses were subsequently further restricted based on participation in each transition, as described below.

### Dietary indicators

Four questions assessed frequency of consumption of fruit, vegetables, sweets/chocolate and sugar-containing soft drinks, based on the World Health Organization (WHO) study on Health Behaviour in School-aged Children (HBSC) [28, 29]. There is no evidence available on the validity of this measure in comparison with other measures of dietary intake. The question stated: “We are interested in knowing how often you usually eat each of the following foods. Consider the last 3 months”. Response categories for fruit, vegetables, and soft drinks were ‘several times a day’ [recoded to 10 times/week], ‘once a day’ [7], ‘3–6 times per week’ [4.5], ‘1–2 times per week’ [1.5], and ‘seldom or never’ [0.5]. Response categories for the sweets/chocolate were ‘every day’ [7], ‘3–6 times per week’ [4.5], ‘1–2 times per week’ [1.5], ‘seldom’ [1], and ‘never’ [0]. At age 30, the response categories for sweets/chocolate were changed so that they matched the response categories for the other three foods. Recoding of the categorical responses to a continuous measure of frequency of consumption per week, as has been done previously [26], facilitated analysis of longitudinal change in intake of each food item. Diet data were excluded where participants reported that they were pregnant (asked at age 23 and age 30 only).

### Life transitions

Five key life transitions have been identified in previous analysis of this cohort: leaving the parental home, leaving education, beginning employment, cohabitation and parenthood [30]. Parenthood is only included as a covariate in this analysis since only a small proportion of individuals (*n* = 295) reported having children by age 30, among whom the majority reported having their first child in their late 20s, when data collection was infrequent. We analysed data on life transitions from age 15 onwards for living situation and from age 16 for occupation. Prior to these ages it was assumed that all participants were attending school and that living situation at age 14 was the same as at age 15. Data from two questions “Who do you live with?” (ages 15 to 30) and “What do you presently do in the daytime?” (age 16) or “What is your current vocational status” (age 18 to 30) were first coded into living situation and occupation categories as shown in Table 1. Participants who reported both studying and employment, were coded as ‘employed’ if they reported full-time work, and as ‘student’ over ‘employed’ if they reported part-time working. Binary transition variables were then generated from these data: ‘Leaving home’: all other living arrangements vs. living with parents, ‘Leaving education’: student vs. not student, ‘Entering employment’: employed vs. not employed, ‘Beginning cohabitation’: living with partner vs. all other living arrangements. To focus on the first time of transition only, data on subsequent returns to the pre-transition state were removed from the analysis dataset. Preliminary analyses to assess degree of overlap between transitions showed Phi correlations ranging from 0.36 to 0.79, with the largest correlation seen for leaving education and entering employment.

**Table 1:** Living situation and occupational status of those included at each age of assessment, NLHBS

	Age (years)						
	15	16	18	19	21	23	30
Number of participants	963	789	779	643	634	627	536
Living situation (%)							
Parent(s)	98.6	72.9	70.6	57.7	28.7	14.4	1.7
Friends or others	1.1	16.6	22.3	24.4	29.0	26.0	6.0
Spouse/partner	n/r	n/r	n/r	9.2	20.8	39.9	74.9
Alone	n/r	n/r	6.9	8.2	14.0	18.8	17.5
Missing	0.3	10.5	0.1	0.5	7.4	1.0	0.0
Occupation (%)							
Student	n/r	83.5	87.9	59.3	57.4	45.5	5.2
Employed	n/r	0.6	8.2	25.7	26.2	42.9	81.3
Other	n/r	5.3	3.7	14.8	9.5	11.6	6.0
Missing	n/r	10.5	0.1	0.3	6.9	0.0	7.5

Footnote: Abbreviations: *n/r* not reported at this age

### Covariates

Sex of participants was reported at baseline. Parents of participants reported their educational level in 1996. Responses were collapsed into three categories: elementary school (no education beyond 9 years of mandatory school), upper secondary school (1–2 years and 3 years of upper secondary school), college/university (less than 4 years and 4 or more years of college/university). If both parents provided data, the highest reported education level was used and if parental information was missing, data on parental education reported by the participants themselves at age 15 were used [31, 32].

At age 30 participants reported whether they had children and the year in which their first child was born. This information was used to determine whether participants had children at each wave of data collection; participants were coded as having children at any given wave if they reported a child born in the year in which data collection took place, or any year prior to this.

### Statistical analyses

Analyses were performed using STATA version 14. Summary data on sex and parental education were compared between those participating in the study at age 14 and age 30, to assess for bias due to cohort attrition. Descriptive data on living situation and occupation are presented at each age, where reported. Data on the four diet indicators, fruit, vegetables, SSBs and confectionery, were analysed at each age to give mean and standard deviation of intake for all included participants. We fit multilevel growth models, using maximum likelihood estimation, to each of the diet indicators to analyse changes in diet with age, adjusting for gender and parental education. Multilevel models nested observations at each age within individuals, making use of all available data on each individual. Assessment of model fit based on likelihood ratio testing found that a quadratic model of diet indicators with age best fit the data.

For transition analyses we included only individuals who had gone through each transition under consideration. We explored the use of fixed effects or random effects multilevel models for transition analyses. Since the random effects assumption, as assessed by the Hausman test [33], was not met after inclusion of available covariates, we chose to use fixed effects models. We first modelled the effect of each transition on each of the diet outcomes, including age and age-squared as covariates to adjust for underlying dietary trends with age (unadjusted growth models). We then added the remaining three transitions and parenthood as time-varying covariates, to generate adjusted models, and performed subgroup analyses by gender. Testing of multicollinearity between transition variables in linear regression models showed that variance inflation factors remained below 5, suggesting that collinearity between transition variables was not a cause for concern in these analyses.

## Results

### Characteristics of the study population

A total of 1100 individuals were included in this analysis, who provided data at any age from age 14 to age 30 years. Of all participants, 54.3% were male and 45.7% were female, with parental education (where reported, *n* = 937): 40.3% college/university, 44.7% secondary school and 14.9% primary school only. Of those included in the sample at age 30, compared to those included at age 14, a greater proportion were female (52.8%, *p* = 0.002) and a greater proportion had parents who were more highly educated (40.9% college/university, 40.0% secondary school and 10.1% primary school vs. 37.1% college/university, 39.1% secondary school and 12.6% primary school (*p* = 0.09)). Table 1 shows data on participants’ living situation and occupation at each age, reporting percentages of participants in each category at each age.

### Dietary trajectories from age 14 to age 30

**Fig. 1:**
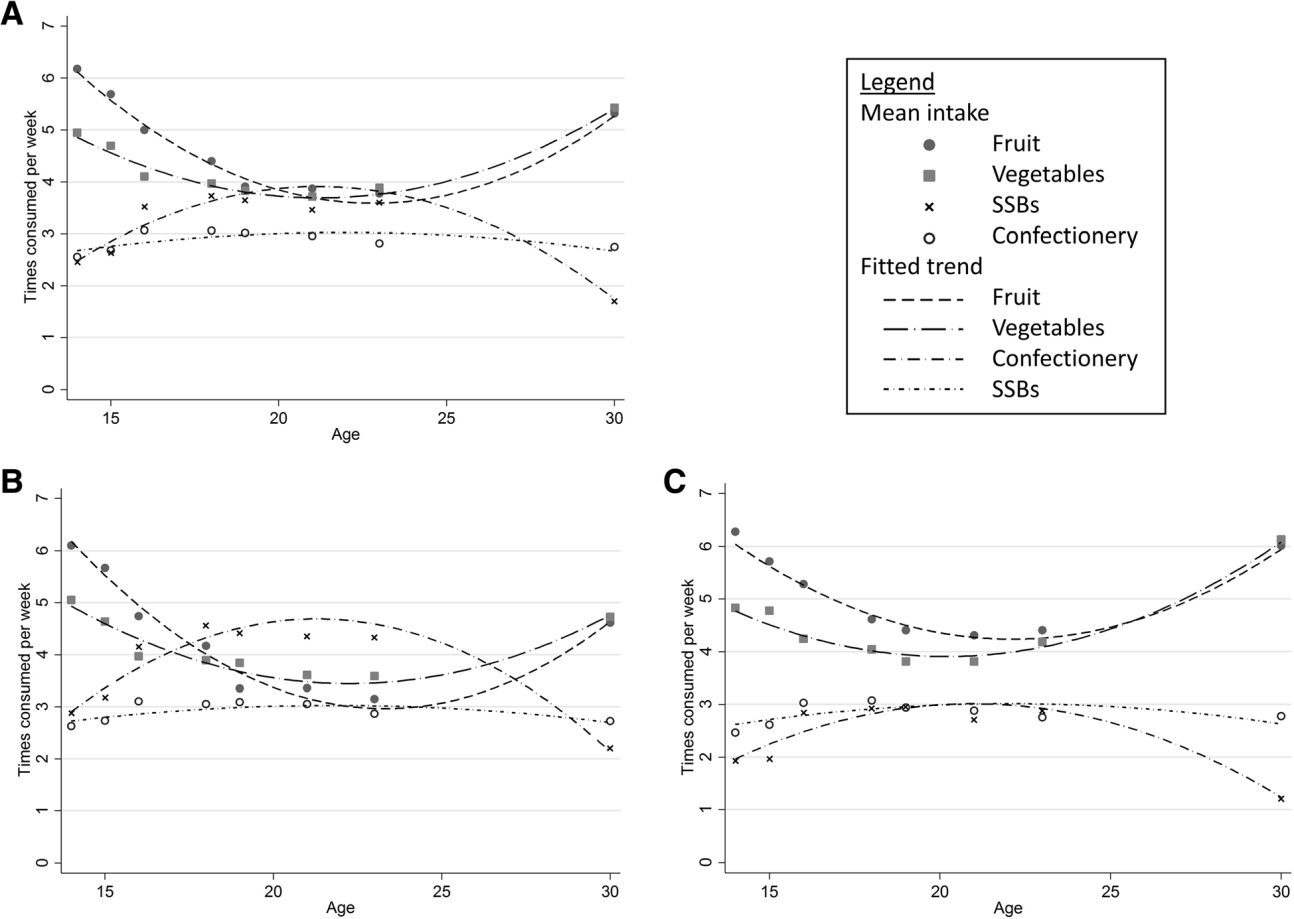
Trajectories of diet from age 14 to age 30, NLHBS. A. Male and female, B. Male only, C. Female only. For underlying data see Additional file 1: Table S1

Figure 1 displays trajectories of change with age across each of the 4 diet variables from age 14 to age 30 (see Additional file 1: Table S1 for mean frequency of consumption at each age). There was a decrease in consumption of fruit and vegetables from age 14 through to the early 20s, followed by an increase to age 30. For example, fruit consumption decreased from 6.2 times per week (SD 3.0) at age 14, to 3.9 times per week (SD 3.2) at age 19, increasing to 5.3 times per week (SD 3.3) at age 30. The opposite pattern was seen for SSBs (2.5 (SD2.1), 3.6 (SD3.0), 1.7 (SD2.2) times per week at ages 14, 19 and 30 respectively) and to a lesser extent for confectionery consumption. A smaller degree of change in diet was seen for females than males.

### Association of life-course transitions with change in diet

Associations between each of the four life-course transitions and four diet indicators are shown in Table 2. Leaving the parental home was the transition which showed the highest association with dietary indicators. This transition was associated with a decrease in fruit and vegetable consumption frequency in both males and females, of around one consumption occasion every 2 weeks. Leaving home was associated with a similar decrease in SSB intake frequency, but among males only in adjusted models. Leaving education was associated with an increase in SSB intake (0.49 times/week) and confectionery intake (0.33 times/week), following adjustment for other transitions, but with a much stronger association for females compared to males when the sexes were analysed separately. Associations between entering employment and beginning cohabitation and diet indicators were limited; beginning cohabitation was associated with an increase in fruit intake in the adjusted model, while entering employment also showed an association with fruit intake in the unadjusted model, but confidence in this association became weaker on adjustment for other transitions.

**Table 2:** Associations of four life-course transitions with weekly intake of fruit, vegetables, confectionery and SSBs, NLHBS

	Both sexes (unadjusted growth models)				Both sexes (adjusted models)			Males (adjusted models)			Females (adjusted models)		
	n	beta	95% CI	*p*-value	beta	95% CI	*p*-value	beta	95% CI	*p*-value	beta	95% CI	*p*-value
Fruit													
Leaving home	792	−0.38	−0.63,-0.12	0.004	−0.54	− 0.87,-0.22	0.001	− 0.49	−0.96, − 0.02	0.04	−0.68	−1.13, − 0.22	0.004
Leaving education	719	0.15	−0.11,0.42	0.26	0.15	−0.31, 0.60	0.52	0.04	−0.62, 0.70	0.90	0.13	−0.51, 0.76	0.70
Entering employment	590	0.35	0.05,0.65	0.02	0.47	−0.11, 1.05	0.11	0.25	−0.51, 1.00	0.52	0.81	−0.09, 1.71	0.08
Beginning cohabitation	522	0.18	−0.16,0.52	0.29	0.54	0.07, 1.01	0.02	0.50	−0.27, 1.27	0.20	0.42	−0.17, 1.02	0.16
Vegetables													
Leaving home	792	−0.46	− 0.68,-0.25	< 0.001	− 0.43	−0.70, − 0.15	0.002	−0.32	− 0.72, 0.08	0.12	− 0.55	−0.92, − 0.18	0.004
Leaving education	719	0.03	−0.20,0.26	0.79	0.28	−0.10, 0.66	0.15	0.40	−0.16, 0.97	0.16	0.06	−0.46, 0.57	0.83
Entering employment	590	−0.19	−0.45,0.06	0.14	−0.31	− 0.79, 0.18	0.22	− 0.53	−1.18, 0.12	0.11	− 0.03	−0.77, 0.70	0.93
Beginning cohabitation	522	−0.15	−0.43,0.14	0.32	0.28	−0.11, 0.67	0.16	0.24	−0.41, 0.89	0.46	0.28	−0.21, 0.78	0.27
Confectionery													
Leaving home	792	−0.06	− 0.23, 0.11	0.50	0.00	−0.21, 0.21	1.00	−0.08	− 0.41, 0.24	0.61	0.05	−0.23, 0.33	0.73
Leaving education	719	0.04	−0.13, 0.22	0.63	0.33	0.04, 0.62	0.03	0.15	−0.31, 0.61	0.52	0.53	0.15, 0.92	0.01
Entering employment	590	−0.15	−0.35, 0.05	0.13	−0.25	− 0.63, 0.13	0.20	− 0.13	−0.66, 0.39	0.62	−0.40	− 0.96, 0.16	0.16
Beginning cohabitation	522	−0.19	−0.41, 0.03	0.10	−0.10	− 0.40, 0.20	0.51	0.01	−0.52, 0.55	0.97	−0.22	− 0.59, 0.14	0.24
SSBs													
Leaving home	792	−0.22	−0.45, − 0.00	0.05	−0.27	− 0.55, 0.00	0.05	− 0.50	−0.94, − 0.06	0.03	0.00	− 0.34, 0.34	0.99
Leaving education	719	0.00	−0.23, 0.24	0.97	0.49	0.10, 0.87	0.01	0.09	−0.54, 0.71	0.78	0.80	0.32, 1.28	0.001
Entering employment	590	−0.11	−0.38, 0.16	0.43	−0.11	− 0.61, 0.39	0.66	− 0.25	−0.97, 0.47	0.49	−0.05	− 0.74, 0.64	0.89
Beginning cohabitation	522	−0.09	−0.38, 0.19	0.52	−0.02	− 0.41, 0.37	0.92	− 0.34	−1.06, 0.38	0.35	0.20	−0.25, 0.65	0.38

Footnote: Fixed effects models are used to look at within-individual change, adjusting for differences between individuals. Unadjusted growth models include age and age-squared as time variables. Adjusted models additionally control for the three remaining life transitions, and for parenthood. Abbreviations: *n* number of participants, *SSBs* sugar-sweetened beverages

## Discussion

### Principal findings

Analysis of changes in consumption of fruit, vegetables, SSBs and confectionery suggest that diet quality declines from adolescence into early adulthood, before improving again by age 30. Within these underlying diet trajectories our findings suggest that, of the lifestyle transitions studied, the most important in relation to change in diet was leaving home, which showed strong negative associations with fruit and vegetable consumption. Leaving education was associated with increases in intake of confectionery and SSBs, with stronger associations seen among females than males. We found limited associations between entering employment or beginning cohabitation and diet indicators.

### Strengths and limitations of the study

This is the first analysis, to our knowledge, to investigate longitudinal associations between multiple life-course transitions and diet in early adulthood. Strengths of this analysis include the use of panel data, which allowed us to study within-person changes with age, without confounding from between-person differences. Data on multiple transitions allowed us to assess associations between diet indicators and individual transitions independently, controlling for co-occurrence of other transitions.

As is frequently the case in large longitudinal datasets, our data only included a small number of self-reported measures of diet. These measures were based on those used in the WHO HBSC study [28, 29] and, as such, have been widely used across a range of different settings; however, their validity when compared to other measures of diet is unknown. Limitations of such methods include their reliance on the individual to estimate their usual intake, and there is no opportunity for adjustment for reporting biases, for example, based on total energy intake. An advantage of this dataset is that the same questions and response items have been used consistently across a long time period, providing an important resource for studying changes in diet over time. In this study, where we analyse association of transitions with within-person variation in diet over time, our measures will be affected by within-person but not between-person variation in reporting error. There is no evidence addressing whether within-person misreporting of diet varies with age, however we believe that systematic bias is unlikely to contribute to the reported associations with life transitions.

The participants of this study were recruited as a representative sample of 13-year-olds from Hordaland County, Norway in 1990. It is unknown how generalizable findings from this population may be across individuals from other regions or time periods. Previous studies have documented differences in adolescent fruit and vegetable consumption between countries [34], as well as secular trends [34, 35]. However, pathways to adulthood seen in this dataset are similar to those seen in the UK and the US [30], and there is no evidence that the impact of the transitions on diet will have changed over time.

This dataset is relatively large with *n* = 1100 participants available for our analysis. However, not all participants went through all transitions, so the numbers included in each transition analysis were lower than this. While this dataset was adequate to study the effect of individual transitions on diet outcomes, the size of the dataset was not sufficient to study further disaggregation of the transition events, interactions between transitions, differential effects of transitions with age, or in subsets of the population beyond sex. Some individuals may have moved between different living arrangements (for example), between waves but then returned to the former status before the next data collection. Such transient transitions would not have been captured in our dataset.

### Comparison with previous evidence and implications of the findings

Across the age range studied we found that mean frequency of fruit and vegetable consumption in this population remained below seven times per week. It is difficult to compare intake frequency data with recommended intakes in grams, however, assuming a maximum of 3 portions (240 g) at each intake occasion, these data suggest that mean intakes were well below the 400 g per day of fruit and vegetables recommended by the WHO [36]. Nevertheless, reported dietary intakes in this cohort are comparable to Scandinavian data from the HBSC surveys, as described previously [26].

Only a small number of previous studies have reported repeated data on food group intakes over time during adolescence and young adulthood [37], making it difficult to assess whether diet trajectories seen in this cohort are consistent with other populations [38, 39]. Several secular factors may have influenced the changes in consumption seen in this cohort. Intake of ‘5 a day’ of fruit and vegetables was introduced as a national recommendation in 1996 [40], when this cohort were aged 19 and fruit and vegetable consumption increased in Norway from 2000 to 2005, with a decrease in soft drink consumption reported over a similar period [35].

Leaving home showed the strongest association with fruit and vegetable consumption in these analyses; a decrease of around 0.5 times consumed per week of both fruits and vegetables suggests a significant impact of this transition on overall diet quality. Given previous evidence to suggest that the home environment and family meals are important predictors of fruit and vegetable consumption [12, 13], it is perhaps unsurprising that a move away from the parental home had detrimental effects on fruit and vegetable consumption. Contributing factors could include the influence of new social or physical environments, but also individual factors such as levels of knowledge, food preparation skills or access to resources; further research is needed to better understand the mediators of this association.

We are not aware of other studies which have investigated dietary changes as individuals leave education. It is interesting that associations between leaving education and increases in sugar-sweetened beverage and confectionery intake only became apparent after control for other transition variables, and are therefore not attributable to other concurrent transitions. Given the correlation between ‘leaving education’ and ‘entering employment’, it may be that associations between leaving education and increased unhealthy food consumption are only seen in those who do not immediately enter employment, which, according to previous analysis, is more common among women than men in this cohort [30]. Others have put forward the ‘Structured Days Hypothesis’ suggesting that children’s behaviour is less obesogenic when their time is structured (i.e. within school) than outside of school [41], and despite little evidence, it is possible such a hypothesis may also apply to young adults.

We were surprised to see limited associations of cohabitation with dietary factors in this sample, given the change in social and physical environment that beginning cohabitation entails and evidence from previous qualitative studies [42, 43]. However, the results from our analyses may reflect that changes observed are not in a consistent direction. More detailed studies have suggested that the direction of behaviour change depends upon the partner’s behaviour [44]. This will be an important question to examine longitudinally in a dataset where such information is available.

Males showed larger changes with age than females when considering overall changes in the four diet indicators. By contrast, changes over transitions were greater in females than males in some instances (in particular the decrease in fruit and vegetable consumption on leaving home and the increase in confectionery and SSB consumption on leaving education). It is unclear why this is the case, although a cross-sectional study among adolescents suggested stronger associations of diet with the physical and social home environment among females than males [12]. It may therefore be that females are on average more responsive to changes in their physical and social environments than males. However further research into the determinants of dietary change, disaggregated by sex, is needed to test this hypothesis.

Acknowledging the limitations of this study, our findings have implications for policy and intervention development. Findings suggest that leaving the parental home is a key time at which individuals could be better supported to prevent a reduction in diet quality. Leaving education may also be an important time to address increases in unhealthy behaviours in females. The “habit discontinuity hypothesis” suggests that behaviour change is more likely to be successful when a context change disrupts individuals’ pre-existing habits [10, 11], and these transition periods may present an opportunity for effective diet and obesity intervention. Further study of these transitions is needed to understand in more detail the different pathways that individuals take in their journey into adulthood, and the mechanisms mediating associations between transitions and changes in diet, to support intervention development.

## Conclusions

Superimposed on the underlying quadratic trends in consumption of fruit, vegetables, SSBs and confectionery, two life transitions, leaving home and leaving education, were associated with negative changes in diet. Leaving home showed strong negative associations with fruit and vegetable consumption, while leaving education was associated with increases in intake of confectionery and SSBs. We found limited associations between entering employment or beginning cohabitation and diet indicators. Leaving home and leaving education may therefore represent times when further intervention is needed to ameliorate decreases in diet quality.

## Additional file

Additional file 1: **Table S1**. Fruit, vegetables, SSBs and confectionery consumed at each age, NLHBS. (DOCX 17 kb)

## Abbreviations

CI: Confidence interval
HBSC: Health Behaviour in School-Aged Children
NLHBS: Norwegian Longitudinal Health Behaviour Study
SSB: Sugar sweetened beverage
WHO: World Health Organization
