# Supplementary material for: Changes in diet through adolescence and early adulthood: longitudinal trajectories and association with key life transitions
Source: Int J Behav Nutr Phys Act. 2018 Sep 10;15:86. doi: 10.1186/s12966-018-0719-8 (PMC6131755; doi:10.1186/s12966-018-0719-8)
Supplement: Supplementary file 1 — Table S1. Fruit, vegetables, SSBs and confectionery consumed at each age, NLHBS. (DOCX 17 kb) [file 12966_2018_719_MOESM1_ESM.docx]

Table S1: Fruit, vegetables, SSBs and confectionery consumed at each age, NLHBS.

|  | **Diet, times consumed per week, mean(SD), at age in years:** | | | | | | | |
| --- | --- | --- | --- | --- | --- | --- | --- | --- |
|  | **14** | **15** | **16** | **18** | **19** | **21** | **23** | **30** |
| **Male and female** | | | | | | | | |
| Fruit | 6.2 (3.0) | 5.7 (3.2) | 5.0 (3.20) | 4.4 (3.2) | 3.9 (3.2) | 3.9 (3.1) | 3.8 (3.1) | 5.3 (3.3) |
| Vegetables | 5.0 (2.8) | 4.7 (2.8) | 4.1 (2.64) | 4.0 (2.7) | 3.8 (2.7) | 3.7 (2.6) | 3.9 (2.6) | 5.4 (2.7) |
| SSBs | 2.5 (2.1) | 2.6 (2.3) | 3.5 (2.80) | 3.7 (3.0) | 3.6 (3.0) | 3.5 (2.9) | 3.6 (3.0) | 1.7 (2.2) |
| Confectionery | 2.6 (1.8) | 2.7 (1.9) | 3.1 (2.04) | 3.1 (2.0) | 3.0 (2.0) | 3.0 (2.0) | 2.8 (1.9) | 2.8 (2.2) |
| **Male** | | | | | | | | |
| Fruit | 6.1 (2.9) | 5.7 (3.2) | 4.7 (3.15) | 4.2 (3.2) | 3.4 (2.9) | 3.4 (2.9) | 3.2 (2.9) | 4.6 (3.1) |
| Vegetables | 5.1 (2.8) | 4.6 (2.8) | 4.0 (2.59) | 3.9 (2.6) | 3.9 (2.7) | 3.6 (2.5) | 3.6 (2.5) | 4.7 (2.5) |
| SSBs | 2.9 (2.4) | 3.2 (2.5) | 4.2 (2.90) | 4.6 (3.0) | 4.4 (3.1) | 4.4 (3.0) | 4.3 (3.0) | 2.2 (2.6) |
| Confectionery | 2.6 (1.8) | 2.7 (1.9) | 3.1 (2.05) | 3.1 (2.0) | 3.1 (2.0) | 3.1 (2.0) | 2.9 (2.0) | 2.7 (2.2) |
| **Female** | | | | | | | | |
| Fruit | 6.3 (3.1) | 5.7 (3.2) | 5.3 (3.23) | 4.6 (3.2) | 4.4 (3.4) | 4.3 (3.2) | 4.4 (3.2) | 6.0 (3.3) |
| Vegetables | 4.8 (2.8) | 4. 8 (2.8) | 4.2 (2.69) | 4.0 (2.7) | 3.8 (2.7) | 3.8 (2.6) | 4.2 (2.8) | 6.1 (2.8) |
| SSBs | 1.9 (1.7) | 2.0 (1.7) | 2.8 (2.51) | 2.9 (2.8) | 3.0 (2.7) | 2.7 (2.7) | 2.9 (2.8) | 1.2 (1.6) |
| Confectionery | 2.5 (1.7) | 2.6 (1.8) | 3.0 (2.03) | 3.1 (2.0) | 2.9 (2.0) | 2.9 (2.0) | 2.8 (1.8) | 2.8 (2.2) |

Footnote: Abbreviations: SSBs, sugar-sweetened beverages
